# Larix Sibirica Arabinogalactan Hydrolysis over Zr-SBA-15; Depolymerization Insight

**DOI:** 10.3390/molecules27248756

**Published:** 2022-12-09

**Authors:** Valentin V. Sychev, Yuriy N. Malyar, Andrey M. Skripnikov, Yuriy A. Trotsky, Yulia N. Zaitseva, Anna O. Eremina, Valentina S. Borovkova, Oxana P. Taran

**Affiliations:** 1 Institute of Chemistry and Chemical Technology SB RAS, Krasnoyarsk Science Center, Siberian Branch, Russian Academy of Sciences, Akademgorodok 50, Bld. 24, 660036 Krasnoyarsk, Russia; 2 Department of Non-Ferrous Metals and Materials Science, Siberian Federal University, pr. Svobodny 79, 660041 Krasnoyarsk, Russia

**Keywords:** arabinogalactan, biomass, hydrolysis, solid acid, catalyst, depolymerization, reaction mechanism, SBA-15, Zr

## Abstract

Arabinogalactan depolymerization over solid Zr-containing SBA-15-based catalyst was studied via HPLC, GPC, and theoretical modeling. Arabinogalactans (AG) are hemicelluloses mainly present in larch wood species, which can be extracted on an industrial scale. The application of solid acid catalysts in the processes of hemicellulose conversion can exclude serious drawbacks such as equipment corrosion, etc. Characterization of 5%Zr-SBA-15 confirmed the successful formation of the mesoporous structure inherent to SBA-15 with fine Zr distribution and strong acidic properties (XRD, XPS, FTIR, pH_pzc_). Carrying out the process at 130 °C allowed us to achieve total products yield of up to 59 wt%, which is represented mainly by galactose (51 wt%) and minor (less than 9 wt%) presence of arabinose, furfural, 5-HMF, and levulinic acid. The temperature increases up to 150 °C resulted in a total product yield drop down to 37 wt%, making temperature elevation above 130 °C obsolete. According to the theoretical investigations, arabinogalactan depolymerization follows the primary cleavage of the β(1→3) bonds between the D-galactose units of the main chain, which is also confirmed by GPC.

## 1. Introduction

The catalytic conversion of lignocellulosic biomass (LCB), as a renewable resource, in recent years, has gained a lot of interest due to the constant diminishing of fossil feedstock and the negative impact on worldwide ecology caused by its extraction and use [1,2,3,4,5,6]. LCB is not only represented by growing plants but by wood and agricultural waste [6,7,8,9,10,11]. The main components of lignocellulosic biomass are cellulose (40–50%), hemicelluloses (25–35%), and lignin (23–33%), some minor amounts of extractives and mineral matter (<5%) are also present [12,13].

There are various approaches to LCB valorization including hydrolysis of cellulose and hemicellulose which yield a pool of molecules, including platform chemicals [14,15,16,17,18].

Arabinogalactans (AG) are hemicelluloses mainly present in larch wood species, which can be extracted on an industrial scale. Arabinogalactan has the potential to become a sustainable feedstock for bio-based product production. Immune-stimulatory properties and other bioactivities of AG are being studied [19].

Arabinogalactan structure is represented by a backbone of β-d-galactopyranose with d-galactopyranose and l-arabinofuranose side chains. The average molar ratio of galactose to arabinose in AG is about 6:1 and the molar mass varies in the range of 20,000–100,000 g/mol [20].

The classic approach to hemicelluloses valorization via hydrolysis implements the use of mineral acids such as H_2_SO_4_ as catalysts and faces serious drawbacks such as equipment corrosion, generated waste treatment, etc. [21,22]. The application of solid acid catalysts in the processes of hemicellulose conversion excludes above stated negative effects and has the potential of becoming one of the key steps in developing the concept of a complex LCB biorefinery [23,24,25].

However, hemicelluloses hydrolysis occurs in hot water at above 100 °C. Traditional solid acid catalysts (silica and aluminum oxides, zeolites, etc.) are not stable in terms of leaching and destruction in such aggressive conditions. Zr-containing catalysts exhibiting good catalytic properties in the hydrolysis and dehydration of cellulose are among the stable solid acid catalysts [22,26].

For liquid phase reactions taking the course over solid catalysts, the textural properties of the latter play an important role. Microporosity of catalytically active solid materials causes reagents transporting limitation to active sites and products backward. Catalyst acidity is another factor to take into account since hemicellulose depolymerization efficiency strongly depends on the presence of acid functionalities [27]. Thus, the designed catalyst must be characterized by a meso-macroporous structure and bear acid functionality.

The Zr incorporation into mesoporous silica matrix SBA-15 is known to produce solid catalysts with strong acidic properties [28]. The distinct acidity is a key to achieving a high catalytic activity in a set of reactions. For example, Zr-SBA-15 materials have found their use in the transesterification of crude palm oil with methanol for biodiesel synthesis [29,30]. Intermolecular Prins reaction (C–C-coupling) between β-pinene and paraformaldehyde with excellent selectivity to terpene alcohol Nopol can be carried out over Zr-SBA-15 [31]. Zr-SBA-15 catalyst was shown to be an effective conversion of levulinic acid and alkyl levulinates to γ-valerolactone (GVL) via catalytic transfer hydrogenation (CTH), also known as Meerwein Ponndorf Verley (MPV) reduction [32]. The high acidity of Zr-SBA-15 also allows researchers to perform one-pot cascade synthesis of GVL from furfural, which includes some acid-driven reactions [33].

The literature lacks studies regarding the catalytic performance of such materials in the processes of hemicellulose valorization.

The aim of this paper is a kinetic study of arabinogalactan depolymerization over the solid Zr-SBA-15 catalyst with degradation mechanism elucidation and product distribution over the reaction course determination.

## 2. Results

### 2.1. Catalyst Characterization

The catalyst was prepared by the co-precipitation method adopted from [34] and described in the Section 3. SBA-15 was synthesized by the traditional method described in [34]. Catalyst study by low-temperature N_2_ adsorption revealed S_BET_ (from 903 to 702 m^2^/g), pore volume (from 1.14 to 0.66 cm^3^/g), and pore diameter (from 63.6 to 57.0 Å) reduction and the slight increase in wall thickness (from 63.9 to 66.4 Å) due to zirconium introduction and deposition within/and on matrix walls (SBA-15 and 5%Zr-SBA-15, respectively) (Table 1).

**Figure 1:**
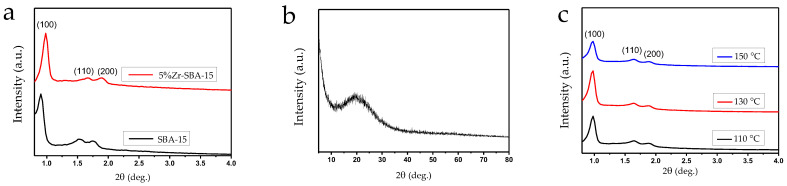
X-ray diffractograms: (**a**) low-angle of SBA-15, 5%Zr-SBA-15, (**b**) wide-angle of 5%Zr-SBA-15, (**c**) 5%Zr-SBA-15 after AG hydrolysis at 110 °C, 130 °C, 150 °C.

X-ray patterns of SBA-15 and 5%Zr-SBA-15 composite catalyst (Figure 1) revealed the presence of the main peaks (100), (110), and (200) corresponding to the successful formation of the mesoporous structure inherent to SBA-15. The shift of the lines to the region of larger angles for zirconium containing sample compared to the pure SBA-15 indicates the localization of zirconium nanoparticles on the matrix walls, which is confirmed by the intensity reduction of the (110) and (200) reflexes [35]. Wide-angle X-ray patterns only reveal a broad peak of amorphous SiO_2_ in the region of 21° reflexes, corresponding to the crystalline phases of zirconium, which were not observed, stating the absence of an aggregated or crystalline state.

Using X-ray fluorescence analysis, the Zr content in obtained composite was determined, revealing close correspondence to theoretical loading (4.5 to 5 wt% percentage respectively) (Table 1).

**Figure 2:**
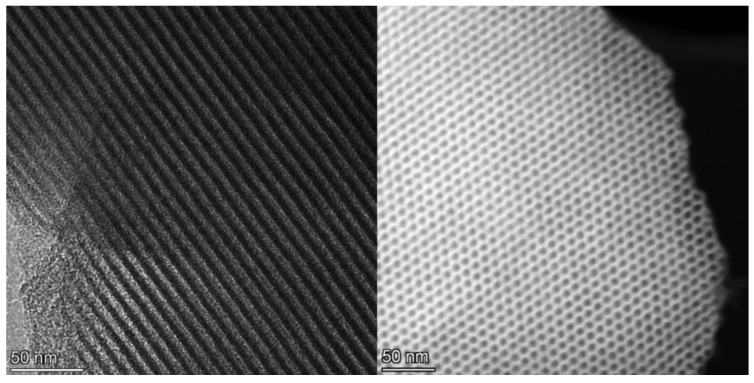
TEM images of 5%Zr-SBA-15.

TEM images of 5%Zr-SBA-15 composite obtained by transmission electron microscopy (Figure 2) revealed the preservation of the honeycomb channel structure, inherent to SBA-15, after Zr introduction.

**Figure 3:**
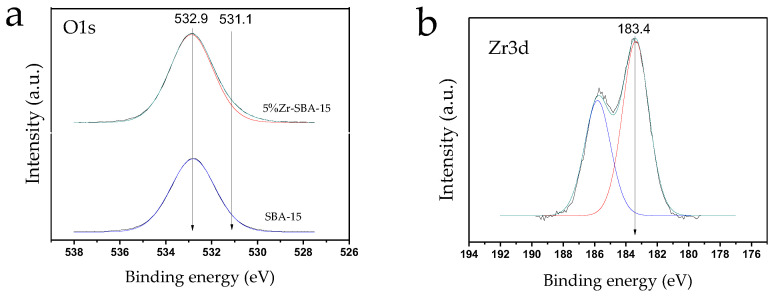
The O1s (**a**) and Zr3d (**b**) XPS spectra of 5%Zr-SBA-15.

Catalyst surface chemical composition and elements’ electronic states were characterized by XPS. It was found that the surface elemental composition corresponds closely to theoretical Zr loading (4.7 to 5 wt%, respectively) (Table 2). The Zr3d_5/2_ spectra reveal the signal at 183.4 ± 0.1 eV typical for the Zr^4+^ state, which corresponds to the ZrO_2_ incorporated within the SBA-15 structure [36,37]. Such signals can be also attributed to zirconium in the ZrSiO_4_ phase (Figure 3) [38].

The O1s spectra of pure SBA-15 and 5%Zr-SBA-15 are characterized by the signals at ~532.9, which correspond to Si^4+^ in SiO_2_. For 5%Zr-SBA-15 the shoulder in the region of lower binding energies (531.1 eV) is present, which can be attributed to the oxygen within zirconium oxide (Figure 3).

**Figure 4:**
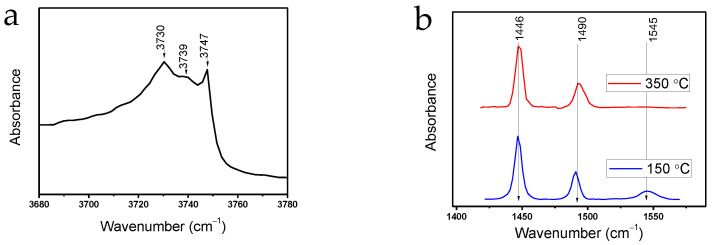
FTIR spectra of 5%Zr-SBA-15 (**a**) before pyridine adsorption, (**b**) desorption of pyridine at 150 and 350 °C.

The acidic properties of 5%Zr-SBA-15 samples were studied using pyridine adsorption coupled with FTIR. The IR spectra of 5%Zr-SBA-15 before pyridine adsorption reveal several absorption bands at 3730, 3739–3741, and 3745–3747 cm^−1^, which correspond to the stretching vibrations of the Si-OH groups (Figure 4). For a pure silicon oxide, one band is usually observed, which corresponds to isolated Si–OH groups [39,40]. The heterogeneity of the OH groups of the studied sample is probably associated with the inclusion of zirconium ions in the silicate framework [41]. Since the diameter of Zr^4+^ ions significantly exceeds the diameter of Si^4+^ ions, upon the substitution in the framework, the Zr–O–Si bond length differs significantly from the Si–O–Si bond length, which leads to structural micro stresses inside the lattice, weakening of the Si-O-Zr–O–H bond, and increasing the Bronsted acidity of silicate materials modified with zirconium [42,43].

Varying the pyridine desorption temperature makes it possible to differ acid sites of different strengths. At 150 °C, pyridine is remained adsorbed on both weak and strong acid sites; with desorption temperature increase to 350 °C, pyridine is retained only on strong sites. After the pyridine desorption at 150 °C, absorption bands are observed at 1446, 1490, and 1545 cm^−1^. The 1545 cm^−1^ band corresponds to the adsorption of pyridine on Bronsted acid sites (BAS) (PyH^+^), the absorption band at 1446–1448 cm^−1^ corresponds to the adsorption of pyridine on Lewis acid sites (LAS), and the absorption band at 1490 cm^−1^ characterizes both LAS and BAS [42,44,45,46]. At 350 °C no bands corresponding to BAS were recorded.

Acid site concentrations calculated from the integral intensities of the characteristic absorption bands using the integral absorption coefficients are provided in Table 3.

Catalyst study via the point of zero charge determination revealed the significant rise of acidity after Zr introduction into the SBA-15 matrix. The pH_pzc_ value of 5%Zr-SBA-15 reached 2.65 when for pure SBA-15 pH_pzc_ 5.20 was measured.

### 2.2. Arabinogalactane Hydrolysis over 5%Zr-SBA-15

Keeping in mind, that the change in the hydrolysis temperature not only affects the overall reaction rate but usually changes the distribution of the final products, the catalytic study was carried out in the range of 110–150 °C allowing us to find the best conditions providing the highest monosaccharides yields and to minimize secondary transformations.

In the study [47], the authors note that the ratio of galactose and arabinose units in larch AG macromolecules varies in the range of 10–12 galactose units per 1 unit of arabinose. As a result, it can be assumed, that the yield of arabinose in the process of hydrolysis will not exceed 8–9 wt%, and that of galactose will not exceed ≈91 wt%.

**Figure 5:**
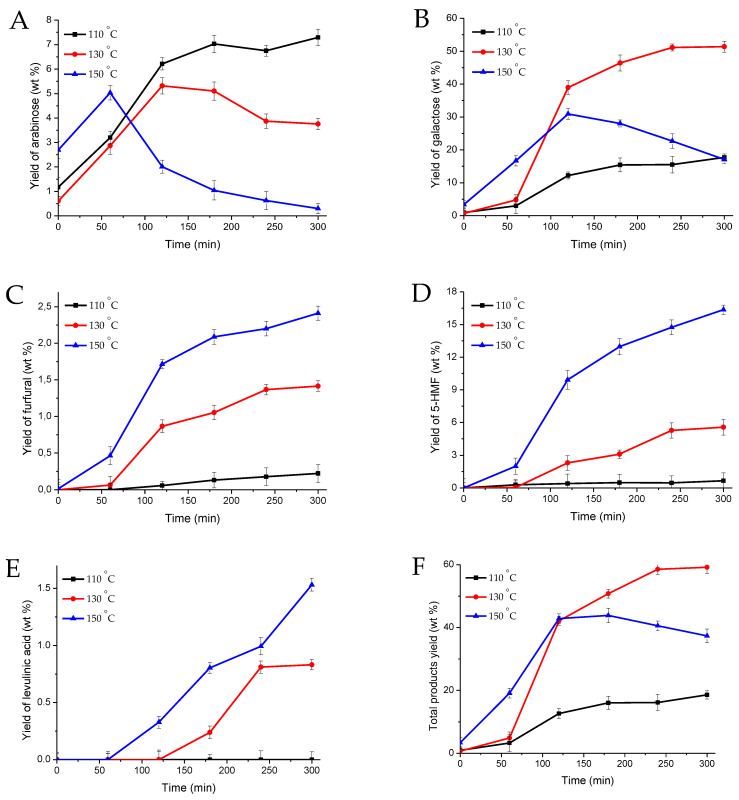
The kinetics of AG hydrolysis over 5%Zr-SBA-15 (n = 3–5). Yield of arabinose (**A**), galactose (**B**), furfural (**C**), 5-HMF (**D**), levulinic acid (**E**), the total yield of products (**F**).

We found out, that a temperature increases from 110 to 150 °C reduces the maximum yield of arabinose from 7.3 wt% for 300 min of the experiment up to 5.0 wt% for 60 min of the hydrolysis of arabinogalactan, due to the dehydration reaction with the formation of furfural (Figure 5). The yield of furfural increases from 0.2 to 2.4 wt% in 300 min of the experiment with an increase in temperature from 110 °C to 150 °C.

The temperature steps up from 110 to 130 °C is followed by a slight rise of products formation reaction rates, TOF and leads to the increase in the maximum yield of galactose from 17.7 to 51.4 wt% within 300 min of the AG hydrolysis process (Table 4, Figure 5). A further increase in temperature to 150 °C is followed by a notable (more than 2-fold increase) rise in products formation reaction rates and TOF value and promotes the intensification of the formation of 5-hydroxymethylfurfural (5-HMF) from galactose (Table 4, Figure 5). As a result, the maximum galactose yield of 30.9 wt% is achieved within 120 min of the experiment, followed by a decrease to 17.5 wt% at 300 min in the process of AG hydrolysis, as well as a significant increase in the content of 5-HMF from 0.2 (110 °C) to 16.4% mass (150 °C).

It should be noted, that at temperatures of 130 and 150 °C, among the reaction products levulinic acid was identified. The maximum yield of which was 0.8 and 1.5 wt%, respectively. Levulinic acid can be produced from both furfural and 5-HMF.

Taking into account obtained data, a conclusion can be made, that AG hydrolysis over 5%Zr-SBA-15 at 150 °C results in excessive saccharides degradation leading to the massive yield drop (more than a half) of galactose and arabinose and drop of total products yields by a third making temperature elevation above 130 °C obsolete. This is presumably due to the formation of humins from furan derivatives, mainly from 5-HMF [48,49]. Regarding the kinetics, we can note, that the release rate of galactose is higher than the one of arabinose. Reaction rates of sugars formation are higher than the ones of sugars acid-driven conversion products (5-HMF, furfural, levulinic acid). Galactose formation is described by the highest reaction rate (Table 4). In addition, the arabinose yields are close to theoretical.

After the AG hydrolysis 5%Zr-SBA-15 was studied by XRD and XRF, to reveal possible structure deterioration over the reaction course. The X-ray diffractograms demonstrate mesoporous structure preservation even after the AG hydrolysis at 150 °C (Figure 1). The XRF data revealed a slight increase in ZrO_2_ content (from 4.5 to 4.6 wt%) after the AG hydrolysis at all temperatures, which can be explained by the leaching of amorphous silica, preserved at the catalyst surface after synthesis but not incorporated into main Zr-SBA-15 matrix.

### 2.3. Gel Permeation Chromatography of Hemicelluloses

**Figure 6:**
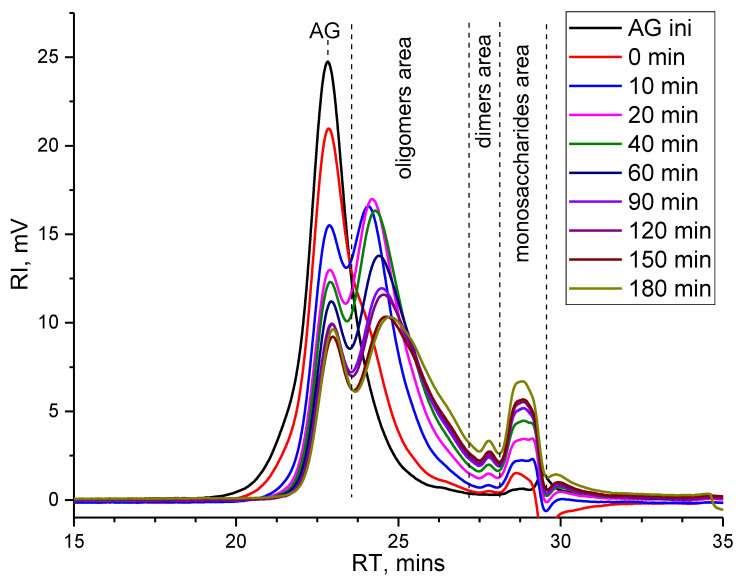
Gel permeation chromatogram of the arabinogalactan catalytic depolymerization products at a temperature of 110 °C.

To elucidate the mechanism of AG hydrolysis over 5%Zr-SBA-15 catalyst, the samples taken during the reaction course at 110 °C were analyzed using GPC [50,51]. The depolymerization of AG begins during the autoclave heat-up to the desired temperature, which explains the presence of a shoulder at the main AG peak (curve 0 min, RT 22.3 min, molecular weight 14 kDa), as well as a peak in the region of 29 min, which attributed mainly to monosaccharides-arabinose and galactose (Figure 6).

Over the reaction course, the native AG molecules portion decreases and the formation of low molecular weight compounds is observed. The nature of molecular mass distribution changes from monomodal to bimodal, and there is a gradual increase in the portion of oligomers and mono- and dimeric products of AG hydrolysis. The greatest signal increase is located in the oligomers region with a molecular weight of around 7 kDa, which indicates the cleavage dominance of the β(1→3) bonds of the main chain, consisting of D-galactose units. The hydrolysis of side chains, represented mainly by β(1→6) bonds, occurs at a lower extent and corresponds only to a slight increase in the portion of dimers, galactose, and arabinose following the increase in the duration of the catalytic depolymerization process, which correlates with HPLC data.

The molecular weights of dissolved substances as a process result are significantly reduced—the weight average MM changes from 14 kDa to 4 kDa and the decrease in the number average molecular weight is barely present. Such a combination results in a significant increase in polydispersity from 1.2 to 5 at 180 min of the reaction course. The low yield of target products and incomplete depolymerization of native AG indicates the low efficiency of the depolymerization over 5%Zr-SBA-15 catalyst under these conditions (110 °C), making the process intensification necessary.

**Figure 7:**
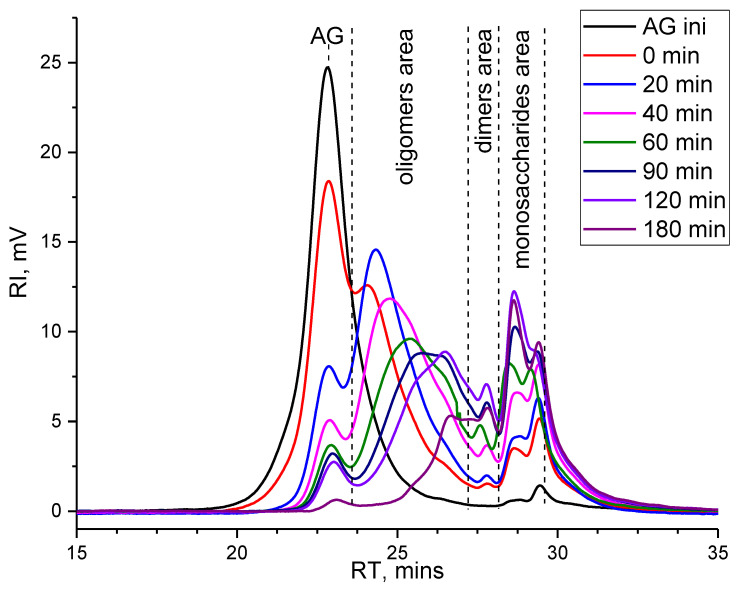
Gel permeation chromatogram of the arabinogalactan catalytic depolymerization products at temperature 130 °C.

To intensify the hydrolysis process, the reaction temperature was raised up to 130°C. The chromatogram corresponding to the process start (Figure 7, curve 0 min,) provides a different understanding of the process course: the peak of native AG decreases, a significant shoulder appears nearby in the oligomeric region (RT 24.5 min), individual peaks of galactose (RT 28.7 min) and arabinose (RT 29.5 min) become visible. Noteworthy, the height and area of the arabinose peak are greater than that of the galactose peak. After 20 min of the process, the peak value of oligomeric depolymerization products becomes larger than native AG, which indicates effective cleavage of β(1→3) bonds between the D-galactose units of the main chain. The chromatogram features suggest the process dominance of the β(1→3) bonds cleavage at this stage—the increase in peaks in the region of mono- and disaccharides is insignificant. Following the reaction time, the destruction of side chains intensifies, which are mainly represented by β(1→6) bonds and leads to further redistribution of the molecular masses of the products to the low molecular weight region. The last stage of the process of catalytic depolymerization of AG is characterized by almost complete depolymerization of native AG, which is clearly represented by a barely visible characteristic peak (Figure 7, curve 180 min). The products are mainly represented by galactose, arabinose, and small molecular weight oligosaccharides. The peak of galactose becomes higher than the peak of arabinose. Nonetheless, the ratio of the areas of these peaks does not correspond to stoichiometry. This confirms the observation made by HPLC that galactose is released more slowly and in a lower yield than arabinose. The absence of peaks with RT over 30 min indicates the absence of monosaccharide degradation processes.

The decrease in the molecular weights of dissolved substances under these process conditions is much more intense—the weighted average MM gradually decreases from 14 kDa to 2 kDa. As the process proceeds, the polydispersity at first increases to 9, which indicates an increase in the content of monosaccharides and oligosaccharides with different degrees of polymerization, then, following the deeper hydrolysis, the composition of the products stabilizes, which results in polydispersity decrease down to 4.

### 2.4. Arabinogalactan Structure Optimization

To understand the mechanism of the depolymerization process, involving the primary cleavage of the β(1→3) bonds between the D-galactose units of the main chain, we modeled the structure of the Siberian larch wood AG. The data on the monosaccharide composition has been widely discussed in the literature [47,52,53]. Based on these data, a model of the AG polysaccharide was developed, representing the main chain consisting of D-galactose units linked by β(1→3) bonds, as well as side branches, represented mainly by β(1→6) linked D- galactose. A small part of the side chains is represented by longer chains with the inclusion of arabinose in the furanose and pyranose form in a ratio of 2:1, as well as a small number of uronic acids, mainly galacturonic. The total ratio of Ara:Gal was 1 to 8, which correlates with the literature data and the molecular weight obtained from GPC.

**Figure 8:**
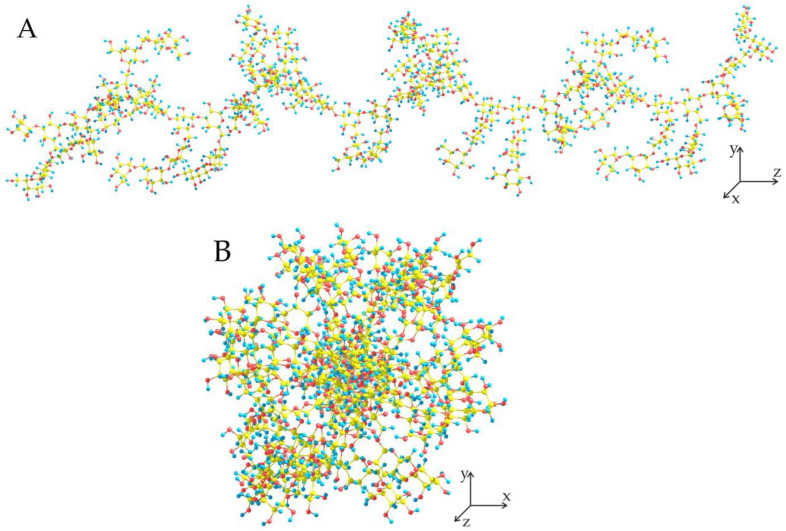
Structure of the arabinogalactan single molecule: (**A**)—X-axis view, (**B**)—Z-axis view.

The AG structure was modeled [54] using the molecular mechanics method [55] via the HyperChem package. Optimization of the geometry of the AG molecule was carried out using the semi-empirical PM6 method in the MOPAC2016 package [56]. The structure of an AG molecule with a mass of about 14 kDa, consisting of 88 units, is shown in Figure 8.

According to the calculations, the AG molecule is a highly branched polysaccharide, the chain length is about 130 Å, and the diameter is 28–35 Å. The main chain of the molecule, consisting of β(1→3) linked D-galactose units, is a “right” helix with a pitch of about 23 Å. Despite the high branching of the AG molecule, the side chains are likely to stabilize with each other, forming intra- and intermolecular hydrogen bonds. At the same time, the main chain is “open” for the action over hydrolysis catalyst, which causes the primary course of the glycosidic bond β(1→3) hydrolysis. It should also be noted that the cleavage of the β(1→3) bond requires less energy than the β(1→6) bond of the side chains, which confirms the course of AG hydrolysis established by GPC [57]. Taking into account the AG molecule size and the catalyst pore diameter (37.6 Å, Table 1) a conclusion can be made, that initial depolymerization occurs on the 5%Zr-SBA-15 surface. Then, the smaller molecule fragments may enter the catalyst pores and undergo further conversions.

Taking into account experimental and theoretical results, we can propose the following mechanism of AG hydrolysis in the presence of a solid acid catalyst. 

Step 1: glycosidic bond β(1→3) hydrolysis.

Step 2: arabinose-galactose bond hydrolysis.

Step 3: galactose-galactose β(1→6) bond of the side chains.

Note, the rate of reactions decreases in the series: w_step1_ > w_step2_ > w_step3_.

## 3. Materials and Methods

### 3.1. Materials

HCl (chemically pure) (Khimreaktivsnab); Pluronic P123 (C_2_H_4_O)_20_(C_3_H_6_O)_70_(C_2_H_4_O)_20_ (M.w.~5860, >98%) (Sigma-Aldrich); TEOS–Si(C_2_H_5_O)_4_, chemically pure (Khimreaktivsnab, Krasnoyarsk, Russia); NH_4_F, analytical grade (Khimreaktivsnab); 70% solution of zirconium propoxide Zr(C_3_H_7_O)_4_ in 1-propanol (Sigma-Aldrich, Burlington, MA, USA); hexane, chemically pure; ZrO(NO_3_)_2_ 2H_2_O chemically pure (Khimreaktivsnab); 25% aqueous ammonia, chemically pure (Khimreaktivsnab). Arabinogalactan (Fibrolar C, Wood Chemistry LLC, Orono, ME, USA) All catalysts and solutions were prepared using water purified on a Milli-Q unit (Millipore, France).

### 3.2. SBA-15 Preparation

SBA-15 synthesis was carried out according to the procedure described in [34]. Ammonium fluoride in the amount of Si:F (mol) = 0.1 was added 5 min before the end of the primary precipitation stage. Hydrothermal treatment (HTT) was carried out at 80 °C for 24 h. After the end of HTT, the material was filtered off and washed with water until the wash water was neutral. The filtrate was dried in the air under conditions close to normal (about 2 days), then at 80 °C for 12 h. The structuring agent was removed from the material by calcination in air at 550 °C (temperature rise rate 3 °C/min).

### 3.3. 5%Zr-SBA-15 Preparation

Synthesis by co-precipitation was carried out according to the procedure [33]. After the administration of TEOS, a solution of Zr(OPr)_4_ was added dropwise, 5 min before the end of the primary precipitation stage, and ammonium fluoride was added in the amount of Si:F(mol) = 0.1. The filtration, washing, drying, and structuring agent removal were carried out similarly to SBA-15.

### 3.4. Catalyst Characterization

Powder diffraction data were obtained using CuKα radiation on an X’Pert PRO diffractometer with a PIXcel (PANalytical) detector equipped with a graphite monochromator. The sample was ground in an agate mortar and prepared by powdering. The surveys were carried out at room temperature in the small-angle range from 0.5 to 5° on the 2θ scale, with a step of 0.026°, ∆t—200 s. In the area of wide angles from 3 to 80° on the 2θ scale, in steps of 0.026°, ∆t is 50 s.

Quantitative elemental analysis of the obtained composite materials was carried out on an AxiosAdvanced X-ray fluorescence spectrometer (PANalytical, Almero, The Netherlands). For analysis, the test material was pressed together with boric acid H_3_BO_3_ as a binder into a tablet with a diameter of 32 mm.

The textural properties of the samples were studied using an ASAP 2420 specific surface analyzer (Micromeritics, Nocross, GA, USA). Degassing was carried out at a temperature of 350 °C; vacuum depth—7 10–6 mm Hg, stage duration—8 h. Registration of nitrogen sorption isotherm at a temperature of 77 K. Isotherms was analyzed to calculate the following textural characteristics: total pore volume determined by the SinglePoint method (VSP), specific pore volume (Vμ), and area (Sμ) of micropores. Pore size distribution curves were calculated by the BJH method [58]. Calculations were made using Micromeritics software.

The XPS study was carried out on a photoelectron spectrometer manufactured by SPECS Surface Nano Analysis GmbH (Germany) using the MgK* experiment (h* = 1253.6 eV). The binding energy scale (Eb) was preliminarily calibrated by the position of the peaks of the core levels Au4f_7/2_ (84.00 eV) and Cu2p_3/2_ (932.67 eV). The samples were applied to a double-sided copper-based conductive adhesive tape. To correct the density photoelectron parameters, an internal standard is used, which is the Si2p line (Eb = 103.5 eV) obtained on the SBA-15 support [59]. Survey spectra and individual spectral regions of Si2p, Zr3d, C1s, and O1s were recorded at an analyzer transmission energy of 20 eV. Determination of the relative content of elements on the surface of the samples and their atomic interactions, measured by the integral resistance of photoelectronic lines, corrected in comparison with the atomic sensitivity coefficients [60]. These data are presented in Table 1. Processing and analysis of income from spectral data are carried out in a specialized program XPS Peak 4.1.

The acidic properties of the catalyst were studied by IR spectroscopy using pyridine as a probe molecule. The spectra were recorded on an IRTracer-100 IR-Fourier spectrometer (Shimadzu, Japan) in the range of 1350–4000 cm^–1^ with a resolution of 4 cm^–1^. The sample was pressed into a tablet 1 × 2 cm^2^ in size, with a total weight of 20–30 mg (the optical thickness of the tablet is equal to the ratio of the mass of the tablet to its area), placed in an IR cell, and calcined in vacuum for 1 h at a temperature of 500 °C and pressure of residual gases not less than 10^−4^ Torr. Next, the sample was cooled to room temperature and its IR spectrum was recorded. Pyridine was adsorbed at 150 °C for 20 min. Next, excess pyridine was desorbed at a given temperature by continuous evacuation for 30 min, the sample was cooled to room temperature, and the IR spectrum was recorded. The pyridine desorption temperature was 150 and 350 °C. The spectra obtained on the absorption scale were normalized to the optical thickness of the tablets. The concentrations of Brønsted and Lewis acid sites (BAS and LAS, respectively) were determined from the integral intensities of the characteristic absorption bands using the integral absorption coefficients: for BAS, absorption bands with a maximum at 1545 cm^−1^ (k = 1.24); for LAS, absorption bands with maxima at 1446–1448 cm^–1^ (k = 1.56) [42,44,45,46].

The total acidity of the catalysts was studied by the technique of determining the point of zero charge (PZC) by the Sørensen de Bruyne method [61]. Ten milliliters of distilled water was added to the potentiometric cell. Then, with continuous stirring, at certain intervals (5–10 min), the test sample was successively added in small portions (0.01 g each) until the values of the glass electrode potential reached constancy [61].

### 3.5. Catalytic Testing

Siberian larch arabinogalactan hydrolysis was carried out at 110–150 °C using a 100 mL autoclave (Autoclave Engineers, Erie, PA, USA) with constant stirring (800 rpm), during the reaction course the samples were taken via outlet. The substrate and catalyst loadings were both 10 g/L. After arabinogalactan and the catalyst introduction into the reactor vessel, distilled water was added (30 mL), then the autoclave was sealed and purged three times with argon. To ensure a convenient sample taking 0.3 MPa of Ar was left in the vessel, then the reaction mixture was then heated.

Arabinogalactan hydrolysis products yields (wt %) were calculated using the formula:

$$Y(\mathrm{wt}\;\%)=\frac{m_{product}}{m_{arabinogalactan}}\times 100\%$$

where Y(wt %) is the yield of the product; *m_product_* (g) is the mass of the product obtained at the end of the reaction; *m_arabinogalactan_* (g) is the initial arabinogalactan loading.

### 3.6. Product Analysis

The weighted average molecular weight M_w_, number average molecular weight M_n_, polydispersity index PDI, and K of the HC samples were determined by GPC using an Agilent 1260 Infinity II multi-detector GPC/SEC system with a refractive detector. The separation was made on two Agilent PL aquagel-OH and one Agilent PL aquagel 30 columns using the solution of 0.1M NaNO_3_ in water with 0.25 g/L NaN_3_ as a mobile phase. The column was calibrated using Agilent polyethylene glycol standards (US). In addition, saccharides standards such as galactose, arabinose, and cellobiose were used for accurate column calibration. The eluent flow rate was 1 mL/min and the sample volume was 100 μL. Before the analysis, the samples were dissolved in the mobile phase (~5 mg/mL) and filtered through a 0.45-μm Agilent PES membrane filter (Millipore). The data collection and processing were performed using the Agilent GPC/SEC MDS software.

Carbohydrates were analyzed using HPLC (Agilent^®^ 1260 Infinity II, RID, Rezex^®^ “RPM-Monosaccharide” 7.8 × 300 mm column; column temperature 70 °C, detector temperature 40 °C, flow 0.6 mL min^−1^, deionized water eluent).

The concentrations of levulinic acid were determined using HPLC Milichrom A-02 (EkoNova, Russia) equipped with a UV detector (registration at λ = 210 nm) and a chromatographic column “Diaspher-250-PA”, 5 μm, 2 × 75 mm (EkoNova, Russia), eluent—(85% 0.075 M LiClO4, 15% ACN), H_2_O.

## 4. Conclusions

As a result of the carried-out study of arabinogalactan depolymerization over the solid 5%Zr-SBA-15 catalyst, a series of conclusions can be drawn.

Via Zr’s introduction into the SBA-15 matrix, we were able to obtain a solid catalyst suitable for an effective liquid phase depolymerization of arabinogalactan.

Characterizing 5%Zr-SBA-15 we confirmed the successful formation of the mesoporous structure inherent to SBA-15 with fine Zr distribution within the silica matrix via XRD and deep interaction of Zr-O-Si species revealed by XPS. The presence of strong acid functionalities was found using pyridine adsorption coupled to FTIR and determining the pH_pzc_ value.

The arabinogalactan hydrolysis was carried out to determine product distribution and depolymerization mechanism using HPLC and GPC. Carrying out the process at 110 °C results in weak AG depolymerization providing only 19 wt% of total product yield. Stepping up the temperature to 130 °C allows for a raised total product yield of up to 59 wt%, which is represented mainly by galactose (51 wt%) and minor (less than 9 wt%) presence of arabinose, furfural, 5-HMF, and levulinic acid within 300 min. At 150 °C, compared to 130 °C, monosaccharides degradation to furfural, 5-HMF, and levulinic acid is notedly distinct. Such temperature increase results in total product yield drop down to 37 wt%, making temperature elevation above 130 °C obsolete.

According to experimental and theoretical results, arabinogalactan depolymerization begins with the primary cleavage of the β(1→3) bonds between the D-galactose units of the main chain. This is followed by the cleavage of bonds between arabinose and galactose. The last step is the hydrolysis of β(1→6) bond between the side chain galactoses.

## Figures and Tables

**Table 1:** Textural and structural properties and elemental composition of Zr-SBA-15 catalyst.

Sample	Cell Parameter a, nm	ZrO_2_ Content, wt. %	Surface Area (S_BET_), m^2^/g	Micropore Surface Area S_µ_ (t-plot), m^2^/g	Pore Volume V_SP_, cm^3^/g	Pore Diameter D_4V/A_, Å	Pore Diameter D (from PSD max), Å	Wall Thickness (W), Å
SBA-15	114.5	0.0	903	327	1.14	50.6	63.6	63.9
5%Zr-SBA-15	104.0	4.5	702	254	0.66	37.6	57.0	66.4

**Table 2:** Catalyst surface chemical composition determined via XPS.

Sample	Si		C		O		Zr		O/Si	Zr/Si
	at.%	wt%	at.%	wt%	at.%	wt%	at.%	wt%		
SBA-15	32.0	43.6	7.1	4.3	60.9	52.1	0.0	0.0	1.90	0.00
5%Zr-SBA-15	29.1	39.3	12.0	7.5	57.9	48.6	1.0	4.7	2.00	0.03

**Table 3:** The acidic properties of 5%Zr-SBA-15.

Catalyst	T_des_	C_BAS_, µmol/g	C_LAS_, µmol/g	pH_pzc_
5%Zr-SBA-15	150	76	223	2.65/5.20 *
	350	-	31	

*—pure SBA-15.

**Table 4:** The initial reaction rates W_0_ (M·sec^−1^ × 10^−3^) * of AG hydrolysis products formation and TOF.

Temp. °C	Arabinose	Galactose	5-HMF	Furfural	Levulinic Acid	TOF **
150	1.57	7.56	1.63	0.479	0.290	4.14
130	1.49	2.25	0.228	0.0659	-	1.29
110	1.35	1.15	0.0371	-	-	1.11

*—$W^{0}=\frac{\Delta_{product}}{\Delta_{t}}$; **—Catalyst activity (TOF) was calculated by the amount of formed products (ν_products_) per mole of Zr according to the equation: $TOF=\frac{\nu_{\mathrm{products}}\;}{\nu_{\mathrm{Zr}}}\;$.

## Data Availability

Data available on request from the authors.

## Sample Availability

Samples of the compounds are available from the authors.
